# On the Diversity-Based Weighting Method for Risk Assessment and Decision-Making about Natural Hazards

**DOI:** 10.3390/e21030269

**Published:** 2019-03-11

**Authors:** Pengyu Chen

**Affiliations:** School of Geography & Resource Science, Neijiang Normal University, Neijiang 641100, China; chenpengyu@njtc.edu.cn; Tel.: +86-0832-2340771

**Keywords:** diversity-based weighting method, entropy-weighting method, variation coefficient method, risk assessment, decision-making, natural hazards

## Abstract

The entropy-weighting method (EWM) and variation coefficient method (VCM) are two typical diversity-based weighting methods, which are widely used in risk assessment and decision-making for natural hazards. However, for the attributes with a specific range of values (RV), the weights calculated by EWM and VCM (abbreviated as *W*_E_ and *W*_V_) may be irrational. To solve this problem, a new indicator representing the dipartite degree is proposed, which is called the coefficient of dipartite degree (CDD), and the corresponding weighting method is called the dipartite coefficient method (DCM). Firstly, based on a large amount of statistical data, a comparison between the EWM and VCM is carried out. It is found that there is a strong correlation between the weights calculated by the EWM and VCM (abbreviated as *W*_E_ and *W*_V_); however, in some cases the difference between *W*_E_ and *W*_V_ is big. Especially when the diversity of attributes is high, *W*_E_ may be much larger than *W*_V_. Then, a comparison of the DCM, EWM and VCM is carried out based on two case studies. The results indicate that DCM is preferred for determining the weights of the attributes with a specific RV, and if the values of attributes are large enough, the EWM and VCM are both available. The EWM is more suitable for distinguishing the alternatives, but prudence is required when the diversity of an attribute is high. Finally, the applications of the diversity-based weighting method in natural hazards are discussed.

## 1. Introduction

It is extremely important to conduct risk assessment and make decisions pertaining to natural hazards in a timely and accurate manner, before the hazards occur. This is of great significance to reduce the possible loss of life and property caused by hazards. At present, there are many methods and techniques for multi-attribute risk assessment and decision-making in natural hazards, such as GIS [1,2,3], TOPSIS [1,4], cluster algorithm [5], artificial neural network (ANN) [6,7], kernel logistic regression (KLR) [8,9] and adaptive neuro-fuzzy inference system (ANFIS) [10], etc. When using some methods and techniques, it is usually necessary to determine the weights of attributes, which affect the reliability of the assessment and decision result. Many methods have been used in determining the attribute weights for natural hazards, such as analytic hierarchy process (AHP) [1,2], the preference ratio method [11], the entropy-weighting method (EWM) [4,5] and the variation coefficient method (VCM) [3,12], etc. The subjective weighting method represented by AHP determines the attribute weights based on experts’ knowledge, but is affected by subjective preference. To avoid the influence of subjective preference on the assessment and decision result, the objective weighting method represented by the EWM should be adopted.

The EWM and VCM are two typical diversity-based weighting methods which calculate attribute weights based on the diversity or dipartite degree of attribute data among the evaluation objects or alternatives [3,4,5,12], effectively avoiding the deviation of human factors [13]. The EWM calculates weights based on the information entropy (IE) of attributes, while the VCM calculates weights based on the coefficient of variation (CV) of attributes. The IE and CV are the indicators to measure the diversity or dipartite degree of attributes. In general, a smaller IE or CV of an attribute indicates a higher diversity or dipartite degree of the attribute, a greater function in risk assessment and decision-making as well as a larger weight of the attribute. For a larger IE or CV, the opposite trends are observed. According to the existing literature, the EWM is more frequently used in determining attribute weights than the VCM. It has been widely used in the risk assessment of various natural hazards, such as floods [4,5], debris flows [14,15,16], drought [17,18] and landslides [19,20], etc. The VCM has been used in the risk assessment of floor water inrush [3,12]. As objective weighting methods, subjective preference is not involved in the EWM or the VCM. To obtain a more reasonable weight, the two methods are always used in combination with subjective weighting methods such as AHP [3,20,21]. Sometimes, the EWM and VCM are even used in combination with each other to determine attribute weights [12].

The EWM and VCM are similar in determining weight because both methods calculate weights based on the diversity of attributes. However, it must be emphasized that there are still some differences between the two methods, as the indicators used to measure the diversity of attributes are different. In this paper, based on a large amount of statistical data, the two methods are compared with each other to analyze the similarities and differences between them. In this paper, it is found that if an attribute has a specific range of values (RV), in some cases both the IE and CV cannot accurately represent the dipartite degree of the attribute, causing the weights calculated by the EWM and VCM (abbreviated as *W*_E_ and *W*_V_) to be irrational. To solve this problem, a new indicator representing the dipartite degree is proposed in this paper, which is called the coefficient of dipartite degree (CDD)—the corresponding weighting method is called the dipartite coefficient method (DCM). A comparison of the DCM, EWM and VCM is carried out in this paper based on two case studies of drought-risk assessment and decision-making for a prevention programme for debris flow. Some suggestions for the application of the three methods in natural hazards are put forward in this paper. This study will provide new insight into the application of information entropy in risk assessment and decision-making for natural hazards.

## 2. Materials and Methods

### 2.1. EWM

The calculation steps of the EWM are described as follows [17,18]:

(1) Establish the evaluation or decision matrix $R=\left\{r_{ij}\right\}$, where $r_{ij}$ is the value of the *j*^th^ attribute in the *i*^th^ object or alternative. Generally, the decision matrix R requires no processing. In some studies, however, the evaluation or decision matrix **R** is normalized [3,22,23,24] and transformed to the normalized matrix $R^{\prime }=\left\{r_{ij}^{\prime }\right\}$, where $r_{ij}^{\prime }$ is the normalized value of the *j*^th^ attribute in the *i*^th^ object or alternative.

(2) Normalize the matrix R or $R^{\prime }$; the calculation equation is written as:

(1) $$F_{ij}=r_{ij}/\sum_{i=1}^{m}r_{ij}\;\mathrm{or}\;F_{ij}=r_{ij}^{\prime }/\sum_{i=1}^{m}r_{ij}^{\prime }$$

where m is the number of objects or alternatives.

(3) Calculate the IE of each attribute by the following equation:

(2) $$E\left(j\right)=-K\sum_{i=1}^{m}F_{ij}lnF_{ij}$$

where E(j) is the IE of the *j*^th^ attribute; K=1/lnm. In particular, when $F_{ij}=0$, let $lnF_{ij}=0$ [17].

(4) Calculate the weight of each attribute by the following equation:

(3) $$w_{j}=\frac{1-E\left(j\right)}{\sum_{j=1}^{n}\left(1-E\left(j\right)\right)}$$

where $w_{j}$ is the weight of the *j*^th^ attribute; n is the number of attributes.

### 2.2. VCM

The standard deviation can be used directly to measure the diversity of attribute data. However, in many cases the attributes have different orders of magnitude and dimensions, and their standard deviations are not comparable, which should be divided by the mean value. Thus, the diversity of the attributes can be comparable. The CV of each attribute can be calculated by the following equation [3]:

(4) $$V\left(j\right)=\frac{\sigma_{j}}{\mu_{j}}$$

where V(j) is the CV of the *j*^th^ attribute; $\sigma_{j}$ is the standard deviation of the *j*^th^ attribute; $\mu_{j}$ is the mean value of the *j*^th^ attribute.

The weight of each attribute can be calculated by the following equation:

(5) $$w_{j}=\frac{V\left(j\right)}{\sum_{j=1}^{n}V\left(j\right)}$$

### 2.3. New Indicator Representing the Dipartite Degree

It is found that in some cases, neither the IE nor the CV can accurately represent the dipartite degree of attribute. When an attribute has a specific RV, the dipartite degree of the attribute is related to its RV. For example, both 10-point and 100-point scoring systems are used in decision analysis. Assuming that there are three alternatives, the scores of an attribute under the 10-point system are 5, 6 and 7; the scores of another attribute under the 100-point system are also 5, 6 and 7. The IEs and CVs of the two attributes are identical, but the dipartite degrees are obviously different. Apparently, the attribute under the 10-point system has a higher dipartite degree. Thus, the RV should be considered when quantifying the dipartite degree of an attribute. Another case is that for three alternatives under the 100-point system, the scores of an attribute are 5, 6 and 7; the scores of another attribute are 95, 96 and 97. The CV of one attribute is 16 times of that of the other attribute, while the 1-IE of one attribute is 257 times of that of the other attribute, but it seems that there is not much difference between the dipartite degrees of the two attributes.

In view of the above two instances, the following new indicator representing the dipartite degree is proposed, which is referred to as the coefficient of dipartite degree (CDD) in this paper. It can be calculated by the following equation:

(6) $$D\left(j\right)=\frac{\sigma_{j}}{L_{j}}$$

where D(j) is the CDD of the *j*^th^ attribute; $L_{j}$ is the size of the RV for the *j*^th^ attribute. For example, the size of the RV under the 100-point system is 100. Obviously, when the RV for each attribute is the same, the standard deviation can be used directly to measure the diversity of the attributes.

In the above two instances, the CDDs are 0.082, 0.0082 and 0.0082, 0.0082, respectively. Obviously, the CDD can represent the dipartite degree of an attribute more accurately.

The weight of each attribute can be calculated by the following equation:

(7) $$w_{j}=\frac{D\left(j\right)}{\sum_{j=1}^{n}D\left(j\right)}$$

In this paper, the method to calculate attribute weights based on the CDD is called the dipartite coefficient method (DCM).

## 3. Results and Discussions

### 3.1. Comparison between EWM and VCM

Statistical data in the literature [3,12,22,23,24,25,26,27] were adopted to calculate the attribute weights using the EWM or VCM, in order to compare the ability of the two methods to accurately determine weight. The referenced studies were selected based on the following principles: (1) the EWM or the VCM was used to calculate attribute weights; (2) raw data were provided. In some studies, the attribute weights were calculated based on normalized data [3,22,23,24]. Correspondingly, when using the other method to calculate weights, normalized data should be adopted.

#### 3.1.1. Similarity

**Figure 1:**
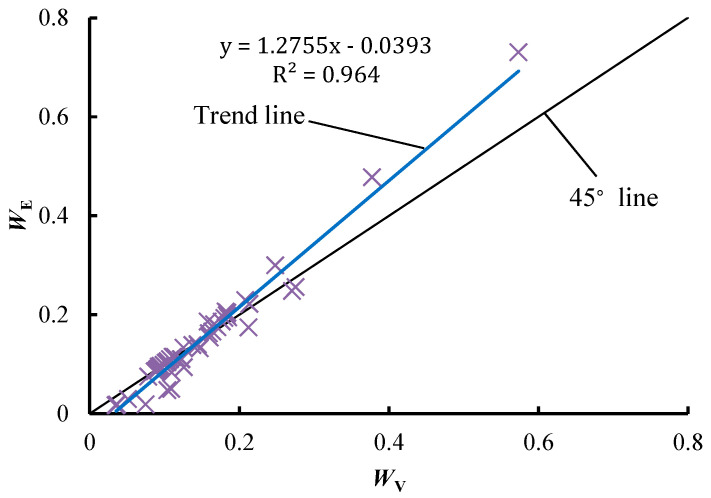
The statistical relation between *W*_E_ and *W*_v_. *W*_E_ and *W*_v_ are calculated using the statistical data in References [3,12,22,23,24,25,26,27].

Figure 1 illustrates the statistical relation between *W*_E_ and *W*_V_. A significant linear relationship is observed between them, and the fitting formula is *y* = 1.2755x − 0.0393, the R^2^ of which is 0.964. This shows a strong correlation between *W*_E_ and *W*_V_. As illustrated in Figure 1, most points are located close to the 45° line (all points on the 45° line have the same values of *W*_E_ and *W*_V_), indicating that these points have almost the same values of *W*_E_ and *W*_V_. However, there are still some points located far away from the 45° line. For these points, *W*_E_ and *W*_V_ are quite different. In particular, *W*_E_ is always larger than *W*_V_ when the diversity of the attribute is high. This difference may result in a quite different decision results between the EWM and VCM.

#### 3.1.2. Difference

**Figure 2:**
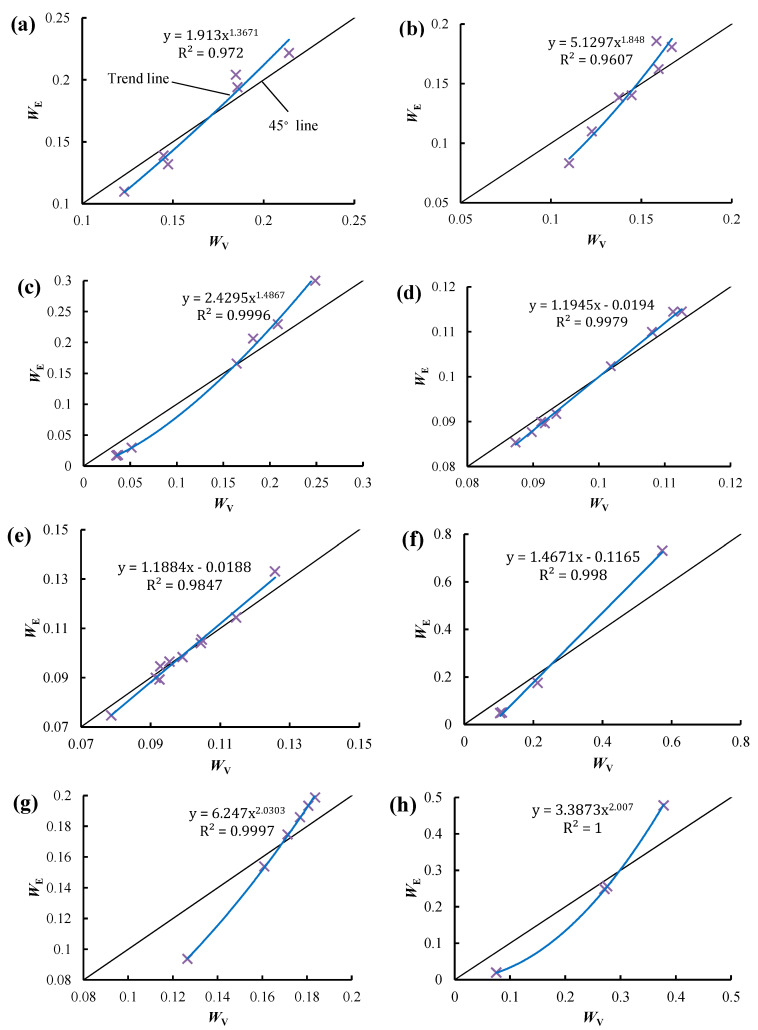
The statistical relation between *W*_E_ and *W*_V_. (**a**–**h**) show the statistical relations obtained based on the statistical data in references [3,12,22,23,24,25,26,27], respectively.

**Figure 3:**
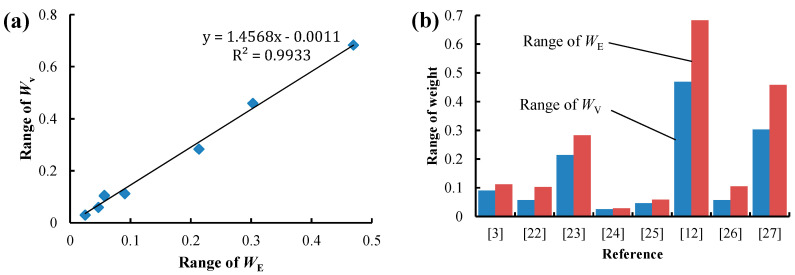
The statistical relation between the range of *W*_E_ and the range of *W*_V_. (**a**) Fitting formula; (**b**) histogram.

Figure 2 illustrates the statistical relation between *W*_E_ and *W*_V_. The two types of weights were calculated using the statistical data in Reference [3,12,22,23,24,25,26,27]. In this paper, exponential function, linear regression, logarithmic function and power function were adopted as fitting functions, and the one with the largest coefficient of determination R^2^ was selected as the final fitting function. A significant linear relationship is observed between *W*_E_ and *W*_V_ in Figure 2d–f, while a significant power function relationship is shown in Figure 2a–c as well as Figure 2g,h. The relationships between the trend line and the 45° line in all the subfigures are similar. This is because when the diversity of an attribute is small, the trend line is located below the 45° line, i.e., *W*_E_ is smaller than *W*_V_. As the diversity of an attribute increases, the trend line is located above the 45° line, i.e., *W*_E_ is larger than *W*_V_. This relationship indicates that the EWM is more sensitive to the diversity of attributes, as the range of *W*_E_ is always larger than that of *W*_V_, as shown in Figure 3a. A positive correlation is observed between the range of *W*_E_ and the range of *W*_V_, and the fitting formula is *y* = 1.4568x − 0.0011, the R^2^ of which is 0.9933, as shown in Figure 3b. The range of *W*_E_ is almost 1.5 times that of *W*_V_, while the mean value of *W*_E_ is equal to that of *W*_V_, indicating that the EWM can better distinguish the weight or diversity of attributes. Correspondingly, the evaluation or decision result is more distinguishable when using the EWM.

When the trend line is located above the 45° line, as the diversity of an attribute increases, the difference between *W*_E_ and *W*_V_ increases, as shown in Figure 2. Thus, the largest *W*_E_ may be much larger than the largest *W*_V_. For example, in Figure 2f, the largest *W*_E_ is 0.730—much larger than the largest *W*_C_ at 0.574. Thus, the evaluation or decision result may be seriously affected by the attribute with the largest *W*_E_, which may result in an irrational evaluation or decision result when using the EWM. This problem will be confirmed in the subsequent case study.

### 3.2. Comparison of DCM, EWM and VCM

#### 3.2.1. Case 1: Drought-Risk Assessment

The IE and CV may not accurately represent the dipartite degree of an attribute with a specific RV, as the dipartite degree of the attribute is related to its RV. To confirm this problem, the drought-risk assessment by Yi et al. [18] was taken as an instance. Observation data [18] and the attribute weights calculated by the EWM, VCM and DCM are listed in Table 1. The three attributes are (1) monthly precipitation anomaly percentage (MPAP), (2) monthly runoff anomaly percentage (MRAP) and (3) monthly soil moisture anomaly percentage (MSMAP), which all have the same RV.

Table 1 shows that the MPAP data vary in a narrow domain [−1, −0] and are concentrated in the ‘‘no drought’’ category. The MRAP and MSMAP data vary in a wide interval [−90, −40] and lie in three grades, namely ‘‘moderate drought’’, ‘‘severe drought’’ and “extreme drought”. As a result, the dipartite degree of MPAP is the lowest among the three attributes [18]. However, the IE of MPAP is zero, and the corresponding weight is 0.9477, which is much larger than that of the other two attributes. This indicates that the dipartite degree of MPAP is the highest among the three attributes. In this case, the weight calculated by the EWM is irrational. Yi et al. [18] believed that the irrational weight is caused by numerous zero values existing in the observation data. These zero values contribute nothing to the IE, resulting in a small IE which cannot accurately represent the dipartite degree of the attribute [18].

To prevent the occurrence of zero values from affecting the weight, some researchers use the translation method to process the observation data [28,29,30]:

(8) $$r_{ij}^{\prime }=r_{ij}+a$$

where a is the translation value, which is usually set to 1 [29,30]; $r_{ij}^{\prime }$ is the value after translation.

Taking MPAP in Table 1 as an example, data are translated to avoid zero values. The translation values are set to −0.1, −1, −10 and −50, as shown in Table 2. The IE, CV and CDD of data after translation are respectively calculated, and the results are shown in Table 2. It can be seen that after data translation by −0.1, the IE of MPAP immediately exhibits a significant change, increasing from 0 to 0.5900. However, it is also smaller than the IEs of MRAP and MSMAP in Table 1, which indicates that the dipartite degree of MPAP is still the highest among the three attributes. After data translation by −1, the IE of MPAP increases to 0.9697, which is smaller than the IE of MRAP and almost equal to the IE of MSMAP. However, this IE still cannot represent the real dipartite degree of MPAP. As the translation value decreases to −50, the IE of MPAP approaches 1, which can represent the real dipartite degree of MPAP. This indicates that as the values of attributes increase, the influence of the RV on the EWM decreases. Although zero values are avoided in data translation, the IE is affected by the translation value, and some IEs may result in an irrational dipartite degree of an attribute if the influence of the RV on the dipartite degree is not considered when using the EWM. Thus, for the attributes with a specific RV, if the values of the attributes are quite small, it is not recommended to use the EWM.

As shown in Table 1, the absolute value of the CV and the corresponding weight of MPAP are the largest among the three attributes, which indicates that the weight calculated by the VCM is also irrational. This is due to the influence of the RV on the dipartite degree, rather than the zero values. Since the mean value will change after translation, the CV varies with the translation value. As the translation value decreases, the CV gradually increases to approach 0, and the real dipartite degree of MPAP gradually emerges, as shown in Table 2. As the values of attributes increase, the mean value approaches the size of the RV, meaning that the influence of the RV on the VCM decreases. Thus, for attributes with a specific RV, if the values of the attributes are quite small, it is not recommended to use the VCM.

As shown in Table 1, the CDD and the corresponding weight of MPAP are the smallest among the three attributes, indicating that its dipartite degree is extremely low, which is consistent with the narrow domain [−1, −0] of the MPAP data. Since the standard deviation will not change after translation, as the translation value decreases, the CDD remains constant (Table 2). This indicates that the CDD is not affected by zero values and translation values. Thus, the CDD can be used to represent the dipartite degree of attributes with a specific RV.

#### 3.2.2. Case 2: Decision-Making for a Prevention Programme for Debris Flow

In the above case study, it is indicated that as the values of attributes increase, the influence of the RV on the EWM and VCM decreases. Thus, if the values of attributes are large enough, the EWM and VCM may be able to determine the weights of attributes with a specific RV. To verify this, the decision-making for a prevention programme for the debris flow hazard in the Sandaogou Mining Area of Fugu County, Shaanxi Province, China [31] was taken as an example. Six attributes for decision-making are “safe reliability”, “environmental harmony”, “economic rationality”, “design standardization”, “construction complexity” and “later maintainability”. The 100-point system is used and experts’ opinions are combined to determine the scores of attributes for each proposed programme [31], as shown in Table 3.

The attribute weights calculated by the EWM, VCM and DCM are listed in Table 4. In this instance, as shown in Table 3, the scores of all the attributes are equal or higher than 70, indicating that the VCM will be only slightly affected by the RVs of the attributes. Thus, the values of *W*_V_ are very close to the weights calculated by the DCM (abbreviated as *W*_D_), as shown in Table 4, resulting in a same optimum prevention programme (Table 5). This indicates that if the values of attributes are large enough, the VCM is able to determine the weights of attributes with a specific RV.

The total score of each proposed programme is calculated by the weighted sum model:

(9) $$S_{i}=\sum_{j=1}^{n}\left(w_{j}r_{ij}\right)$$

where $S_{i}$ is the total score of the *i*^th^ programme; $r_{ij}$ is the score of the *j*^th^ attribute for the *i*^th^ programme; $w_{j}$ is the weight of the *j*^th^ attribute.

The optimum prevention programme is determined based on the ranking of total scores. The total scores and rankings of the proposed programmes are listed in Table 5. As shown in Table 5, the optimum prevention programme determined by the VCM is the same as that determined by the DCM, but different from that determined by the EWM. This depends on the similarities and differences among the attribute weights calculated by the three methods. The EWM was more sensitive to the diversity of attributes, so the rang of the total score of this method was higher than that of the other two methods, as shown in Table 5.

**Figure 4:**
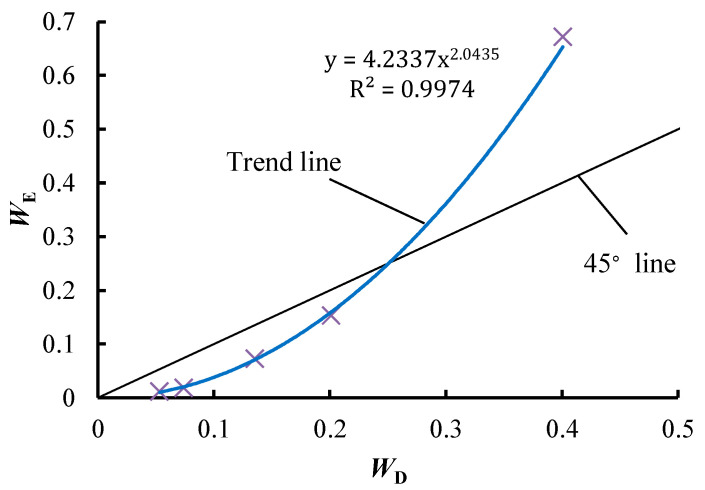
The relationship between *W*_E_ and *W*_D_.

A power function relationship is observed between *W*_E_ and *W*_D_, and the fitting formula is *y* = 4.2337x^2.0435^, the R^2^ of which is 0.9974, as illustrated in Figure 4. This shows a strong correlation between *W*_E_ and *W*_D_, which indicates that the values of *W*_E_ are reasonable. Therefore, if the values of attributes are large enough, the EWM is also able to determine the weights of the attributes with a specific RV. Owing to the power function relationship, *W*_E_ will be much larger than *W*_D_ when the diversity of an attribute is high. For example, the *W*_E_ of “safe reliability” is 0.6715, which is much larger than the *W*_D_ of this attribute (0.4008). Compared to the other attributes, the *W*_E_ of “safe reliability” is too large. The values of *W*_E_ for the attributes of “safe reliability” and “later maintainability” account for over 80% of the total weight, meaning that the decision result almost exclusively depends on these two attributes. For example, the scores of the two attributes for Programme 4 are the highest among all programmes (Table 3), and correspondingly Programme 4 achieves the highest total score when using the EWM, as shown in Table 5.

For the DCM, “safe reliability” still has the largest weight, followed by “later maintainability”; however, the values of *W*_D_ of these two attributes just account for 60% of the total weight, while the values of *W*_D_ of “economic rationality” and “construction complexity” account for 27% of the total weight, which significantly affects the decision result. Owing to the high scores of “economic rationality” and “construction complexity”, Programme 3 achieves the highest total score when using the DCM, even though the scores of “safe reliability” and “later maintainability” for Programme 3 are not the highest among all the programmes.

As shown in Table 3, for “environmental harmony” and “later maintainability”, the scores of Programme 3 are respectively 3 and 5 points lower than those of Programme 4, but for “economic rationality” and “later maintainability”, the scores of Programme 3 are both 10 points higher than those of Programme 4. For other attributes, there is no significant difference in the scores. In terms of the scores of attributes, it seems that Programme 3 is preferred, which is consistent with the decision result of the DCM rather than that of the EWM. This confirms the problem of the EWM mentioned in Section 3.1.2. Compared to the VCM and DCM, the EWM is more suitable for distinguishing alternatives due to its sensitivity to the diversity of attributes. However, when the diversity of an attribute is too high, its decision result may be seriously affected by this attribute, which may result in an irrational decision.

### 3.3. Discussions

As traditional diversity-based weighting methods, the EWM and VCM may be not able to determine the weights of attributes with a specific RV. In this study, a novel diversity-based weighting method called the DCM is proposed to solve this problem. A comparison of the DCM, EWM and VCM is carried out in this paper based on two case studies. The comparison results show that the DCM is preferred for determining the weights of attributes with a specific RV; however, if the values of attributes are large enough, the EWM and VCM are both acceptable. Compared with other weighting methods, the diversity-based weighting methods are more sensitive to the diversity of attributes, which leads to a higher dipartite degree of the decision or evaluation result. In particular, the EWM is more suitable for distinguishing the alternatives than the VCM and DCM due to its high sensitivity to the diversity of attributes. As shown in Table 5, the range of the total score is the highest when using the EWM. However, when the diversity of an attribute is too high, the decision result may be seriously affected by this attribute when using the EWM, which may result in an irrational decision. Therefore, before applying a diversity-based weighting method, we should check whether the attributes have a specific RV as well as check the diversity of attributes, so as to select an appropriate method for accurate weighting results.

When using the diversity-based weighting method, the weight is calculated based on the diversity or dipartite degree of an attribute. For decision-making, this kind of method is suitable for distinguishing alternatives—especially the EWM. For natural hazard risk assessment, an important assumption is made, which is that the dipartite degree of an attribute can represent its importance correctly [18]. However, it was found by Yi et al. [18] that when the observation data are concentrated in the worst category, the dipartite degree of an attribute cannot represent its importance correctly in drought risk assessment. The dipartite degree of an attribute just depends on the statistical data of the attribute, but ignores the relationship between the natural hazard risk and its associated attributes. The formation mechanism of a natural hazard is not considered in the diversity-based weighting method.

Taking debris flow risk assessment as an example, the formation of debris flow is closely related to the condition of material sources, rainfall and topography. An intense rainfall is usually the trigger of debris flow [32,33]. It is obvious that rainfall should be an important attribute in debris flow risk assessment [34]. If the assessed gullies are in the same geographical area, such as a village or a county, there may be little difference in rainfall among the gullies, which indicates that the dipartite degree of this attribute is low. Thus, the weight of rainfall calculated by the diversity-based weighting method will be small. For example, the weight of rainfall calculated by Wang and Sun [15] using the EWM was just 0.051, and that calculated by Wang and Yin [16] using the same method was 0.081; both weights were the smallest among all the attributes. In these cases, the dipartite degree of rainfall cannot represent its importance correctly. Therefore, in natural hazard risk assessment, prudence is required when using the dipartite degree of an attribute to represent its importance. The subjective weighting method determines attribute weights based on experts’ knowledge, which can, to some extent, represent the importance of attributes. It is recommended to use the diversity-based weighting method in combination with a subjective weighting method for risk assessment in natural hazards.

As weighting methods, the EWM, VCM and DCM need to be combined with multi-attribute decision-making or evaluation methods such as the weighted sum model [3], TOPSIS [4] or the cluster algorithm [5] for risk assessment and decision-making in natural hazards. Diversity-based weighting methods do not involve data distribution and correlation between attributes in calculating weights, and do not need to check the independence of each attribute. If the independence of attributes is poor, which will affect the decision or evaluation results, the EWM and VCM can be used combined with principal component analysis (PCA) [35], factor analysis [36] or other similar methods.

## 4. Conclusions

The common diversity-based weighting methods (the EWM and the VCM) for multi-attribute evaluation and decision-making are compared with each other, and a new indicator (CDD) representing the dipartite degree is proposed in this paper. The following conclusions are drawn:

(1) Significant linear and power function relationships are observed between *W*_E_ and *W*_V_, which indicates that there is a strong correlation between them. *W*_E_ and *W*_V_ are usually close to each other, but in some cases the difference between them is large. Especially when the diversity of an attribute is high, *W*_E_ may be much larger than *W*_V_, which may result in an irrational decision when using the EWM. Compared to the VCM, the EWM is more sensitive to the diversity of attributes, as the range of *W*_E_ is always larger than that of *W*_V_.
(2) The IE and CV may not accurately represent the dipartite degree of an attribute with a specific RV, as the dipartite degree of an attribute is related to its RV. The DCM is preferred for determining the weights of attributes with a specific RV, as the CDD can represent the dipartite degree of this kind of attribute correctly.
(3) If the values of attributes are large enough, the EWM and VCM are both able to determine the weights of attributes with a specific RV. Compared to the VCM and DCM, the EWM is more suitable for distinguishing the alternatives due to its sensitivity to the diversity of attributes. However, when the diversity of an attribute is too high, its decision result may be seriously affected by this attribute, which may lead to an irrational decision result.
(4) In natural hazards risk assessment, the dipartite degree of an attribute may not accurately represent its importance; thus, prudence is required when using the dipartite degree of an attribute to represent its importance. It is recommended to use the diversity-based weighting method in combination with a subjective weighting method for risk assessment in natural hazards.

## Figures and Tables

**Table 1:** Observation data and calculated results.

	Attribute		MPAP	MRAP	MSMAP
Observation data	Watershed 1		0 (no drought)	−40 (moderate drought)	−40 (moderate drought)
	Watershed 2		0 (no drought)	−55 (severe drought)	−45 (moderate drought)
	Watershed 3		0 (no drought)	−70 (severe drought)	−60 (severe drought)
	Watershed 4		0 (no drought)	−85 (extreme drought)	−85 (extreme drought)
	Watershed 5		−1 (no drought)	−90 (extreme drought)	−90 (extreme drought)
	RV		[−100, 0]	[−100, 0]	[−100, 0]
Calculated results	EWM	IE	0.000	0.9756	0.9692
		*W*_E_	0.9477	0.0231	0.0292
	VCM	CV	−2.0000	−0.2736	−0.3179
		*W*_V_	0.7718	0.1056	0.1227
	DCM	CDD	0.0040	0.1860	0.2035
		*W*_D_	0.0102	0.4727	0.5171

**Table 2:** Translated data and calculated results.

	Attribute (Translation Value)	MPAP	MPAP (−0.1)	MPAP (−1)	MPAP (−10)	MPAP (−50)
Translated data	Watershed 1	0	−0.1	−1	−10	−50
	Watershed 2	0	−0.1	−1	−10	−50
	Watershed 3	0	−0.1	−1	−10	−50
	Watershed 4	0	−0.1	−1	−10	−50
	Watershed 5	−1	−1.1	−2	−11	−51
Calculated results	IE	0.0000	0.5900	0.9697	0.9995	1.0000
	CV	−2.0000	−1.3333	−0.3333	−0.0392	−0.0080
	CDD	0.0040	0.0040	0.0040	0.0040	0.0040

**Table 3:** Scores of attributes for the proposed programmes [31].

Attribute	Programme 1	Programme 2	Programme 3	Programme 4
Safe reliability	75	70	94	95
Environmental harmony	84	86	82	85
Economic rationality	88	85	90	80
Design standardization	90	94	95	95
Construction complexity	88	85	90	80
Later maintainability	85	80	90	95

**Table 4:** Calculated results of the three methods.

Attribute		Safe Reliability	Environmental Harmony	Economic Rationality	Design Standardization	Construction Complexity	Later Maintainability
EWM	IE	>0.9935	0.9999	0.9993	0.9998	0.9993	0.9985
	*W*_E_	0.6715	0.0116	0.0728	0.0183	0.0728	0.1530
VCM	CV	0.1335	0.0176	0.0439	0.0220	0.0439	0.0639
	*W*_V_	0.4110	0.0540	0.1352	0.0679	0.1352	0.1967
DCM	CDD	0.1115	0.0148	0.0377	0.0206	0.0377	0.0559
	*W*_D_	0.4008	0.0532	0.1354	0.0741	0.1354	0.2010

**Table 5:** Total scores and rankings of the proposed programmes.

Proposed Programme		Programme 1	Programme 2	Programme 3	Programme 4
EWM	Total score	78.801	74.339	92.685	92.701
	Ranking	3	4	2	1
VCM	Total score	81.987	78.517	91.551	90.403
	Ranking	3	4	1	2
DCM	Total score	82.122	78.703	91.548	90.405
	Ranking	3	4	1	2
